# Diagnostic accuracy and clinical performance of deep learning models for grading diabetic retinopathy: a systematic review and meta-analysis

**DOI:** 10.3389/fendo.2026.1853785

**Published:** 2026-07-15

**Authors:** Xin Yan, Shiqi Lei, Lifen Hu, Mu Qin, Na Wu

**Affiliations:** 1 Department of Ophthalmology, The Affiliated Hospital(Clinical College) of Xiangnan University, Xiangnan University, Chenzhou, China; 2 Department of Medical Affairs, The Affiliated Hospital(Clinical College) of Xiangnan University, Xiangnan University, Chenzhou, China

**Keywords:** artificial intelligence, automated grading, deep learning, diabetic retinopathy, meta-analysis, systematic review

## Abstract

**Background:**

Diabetic retinopathy (DR) is a leading cause of preventable visual impairment worldwide, and its precise severity grading is critical for optimizing clinical management. Conventional frameworks, notably the International Clinical Diabetic Retinopathy (ICDR) scale, are often hindered by substantial inter-observer variability and high dependency on specialist expertise. While deep learning (DL) has recently emerged as a transformative approach for automated stratification, a comprehensive synthesis of evidence regarding its diagnostic performance and clinical application remains lacking.

**Objective:**

This systematic review and meta-analysis aimed to comprehensively assess the diagnostic accuracy of fundus image-based deep learning models in the grading of diabetic retinopathy.

**Methods:**

PubMed, Embase, Web of Science, and the Cochrane Library were systematically searched for relevant studies published up to October 28, 2025. Diagnostic accuracy studies utilizing DL algorithms alongside ICDR criteria for diabetic retinopathy grading were included. Literature screening and data extraction were performed independently by two researchers, and the risk of bias was assessed using the QUADAS−2 tool.

**Results:**

A total of 41 studies were included, encompassing various DL architectures and multiple public and private fundus image datasets. In the five-class classification task based on ICDR criteria, the pooled sensitivities of DL-based models varied significantly across severity levels: 95.19% (95% CI: 93.00%–97.00%) for no DR (stage 0), 72.06% (95% CI: 62.06%–81.09%) for mild NPDR (stage 1), 84.33% (95% CI: 78.90%–89.10%) for moderate NPDR (stage 2), 75.84% (95% CI: 68.42%–82.57%) for severe NPDR (stage 3), and 78.82% (95% CI: 71.76%–85.13%) for PDR (stage 4). In the simplified four-class classification task, sensitivities markedly improved across all grades: 96.85% (95% CI: 90.18%–99.93%) for stage 0, 92.94% (95% CI: 79.50%–99.72%) for stage 1, 92.75% (95% CI: 79.31%–99.61%) for stage 2, and 88.19% (95% CI: 68.99%–98.93%) for stage 3.

**Conclusion:**

DL exhibits high sensitivity and substantial potential for DR grading, particularly in screening for no DR and vision-threatening DR. Nevertheless, precisely differentiating between adjacent non-proliferative stages remains a clinical challenge. The observed heterogeneity underscores the imperative for methodological standardization, rigorous external validation, and multimodal data integration. Future research should prioritize enhancing clinical utility and generalizability to facilitate their translation into real-world clinical practice.

**Systematic Review Registration:**

https://www.crd.york.ac.uk/PROSPERO/, identifier CRD420261338867.

## 1 Introduction

Diabetic retinopathy (DR), as one of the most common microvascular complications of diabetes mellitus, has emerged as a public health concern that cannot be ignored worldwide. According to epidemiological data, approximately 25% of global patients with type 2 diabetes are complicated by retinopathy (1). With the continuous rise in the prevalence rate of diabetes mellitus, the disease burden of DR is shifting regionally in a marked trend, spreading from high-income countries to low- and middle-income regions. Of note, the annual incidence rate of DR varies considerably across different populations, ranging from 2.2% to 12.7%, while the rate of disease progression is between 3.4% and 12.3%, among which patients with milder baseline conditions paradoxically face a higher risk of progressing to proliferative DR (2). These epidemiological characteristics are closely associated with metabolic factors such as the level of glycemic control and disease duration, with the impact of glycated hemoglobin (HbA1c) control differences on the prevalence rate of DR being particularly significant (3). Furthermore, socioeconomic factors, including education level and household income, have also been confirmed to be associated with the onset and progression of DR, highlighting the complexity and multidimensional influencing factors of DR (4).

As the grading of DR advances, its treatment strategies exhibit a distinct stepwise pattern. In the non-proliferative diabetic retinopathy (NPDR) stage, strict control of blood glucose and blood pressure remains the fundamental therapeutic approach, whereas anti-vascular endothelial growth factor (VEGF) agents, such as ranibizumab and aflibercept, have become the first-line treatment for diabetic macular edema (DME). However, clinical observations reveal that approximately 40% of patients with DME respond poorly to anti-VEGF therapy, prompting researchers to explore novel treatment strategies targeting inflammatory pathways and neuroprotection (5). For high-risk proliferative diabetic retinopathy (PDR), pan-retinal photocoagulation (PRP) retains its significant role. However, the side effects of PRP, including visual field defects and night vision impairment, have driven the application of modified techniques such as micro-pulse laser. Notably, recent studies have found that retinal neurodegeneration may occur earlier than microvascular lesions, providing a theoretical basis for the development of early interventions, including neurotrophic factors and anti-apoptotic drugs (6). In terms of therapeutic concepts, current research is shifting from simple vascular protection toward a holistic ‘neurovascular unit’ protection strategy, with anti-inflammatory therapies, such as those targeting TNF-α and IL-1β, showing promising application prospects. Of particular note, the concept of ‘ceramidosis’ proposed in recent research has successfully reversed the pathological process of DR in animal models through anti-ceramide immunotherapy, pointing out a new direction for systemic treatment (7).

The grading system for DR has evolved from simple to more complex frameworks. Currently, the internationally recognized gold standard is based on the International Clinical Diabetic Retinopathy Disease Severity Scale. This scale divides DR into five main stages: no DR, mild NPDR, moderate NPDR, severe NPDR, and PDR, with detailed definitions of the characteristic lesions at each stage (8). However, with advances in imaging technology and a deeper understanding of DR, the limitations of this traditional grading system are being observed. Specifically, the grading is based primarily on structural changes, while neglecting functional alterations such as diabetic retinal neurodegeneration, which may exist before the onset of visual impairment. The advent of artificial intelligence (AI)-assisted diagnostic systems has also introduced new requirements for traditional grading. Research on DR grading based on fundus images is undergoing a paradigm shift from traditional manual assessment to AI-assisted diagnosis. Breakthrough progress has been achieved by deep learning (DL) algorithms in the field of automated DR grading (9). The developed convolutional neural network achieved excellent area under the curve (AUC) values for detecting DR requiring referral, with sensitivity and specificity reaching 90.3% and 98.1%, respectively, further validating the efficacy of AI systems in identifying vision-threatening DR, with a sensitivity as high as 95.1% (10).

In recent years, significant advances have been achieved in the fields of explainability, federated learning, and clinical translation within biomedical AI, providing significant insights for in-depth research on DR grading. In terms of explainability, Banerjee et al. have systematically compared dual-branch convolution and attention-driven methods in skin cancer detection, where the degree to which the model focuses on lesion regions was visualized using Grad-CAM and visual attention mechanisms, significantly enhancing clinicians’ understanding of and trust in AI decisions (11). In the classification of brain tumors, PABT-Net, through pyramid attention mechanisms and T-block partitioned feature extraction, not only increases classification accuracy to 99.12% but also enhances the discriminative ability for tumor-specific spatial features, thereby providing architectural support for explainability (12). In the field of federated learning, the FOLC-Net proposed by Khan et al. demonstrates the potential of federated learning in multi-center MRI diagnosis, enabling the collaborative training of high-performance models without the need for data to leave individual hospitals, thus offering a feasible technical pathway for resolving the contradiction between cross-institutional data sharing and privacy protection in DR grading (13). Regarding clinical translation, the UIGO model, which integrates U-Net with Inception modules and incorporates a gravitational optimization algorithm, achieves 99.93% accuracy in the segmentation of liver tumors while maintaining low computational overhead (14). TrionixNet, through an N-core multi-head attention mechanism and multi-scale feature fusion, attains a Dice coefficient of 0.9862 in prostate cancer segmentation and systematically validates the contribution of each module via ablation experiments (15). The ESMXO optimization algorithm exhibits cross-magnification robustness in the classification of breast cancers (accuracy ≥0.947 at magnifications from 40× to 400×), suggesting that it is feasible to adapt DL-based models to different imaging conditions (16). These advances provide significant methodological references for investigating DL in the clinical translation of DR grading in this study.

Nevertheless, these technological advances have also aroused considerable controversy. Studies have found that, in the absence of fluorescein fundus angiography, the accuracy of NPDR grading in affected patients is significantly insufficient, with low diagnostic consistency for moderate NPDR (17). Existing AI algorithms mostly rely on image-level annotations and fail to fully exploit fine-grained information on lesions. Although the framework developed by those AI algorithms, which fuses lesion-level and image-level annotations, has increased grading accuracy, it is still confronted with challenges related to image quality and distribution shifts. Furthermore, the performance of different algorithms in DR grading varies substantially, suggesting that it is necessary to establish more unified assessment standards (16). At present, there remains a lack of systematic evidence summarizing the current status of the applicability of DL in detecting DR grading and quantifying associated accuracy. Therefore, this study aims to systematically assess the current status of the applicability of DL in detecting DR grading, to quantitatively assess the accuracy of DL in detecting DR grading, and to provide evidence−based support for establishing AI−specific standards for diagnostic reporting.

## 2 Methods

### 2.1 Registration

This study was performed in accordance with the Preferred Reporting Items for Systematic reviews and Meta-Analyses 2020 (PRISMA 2020) guideline (**Supplementary Material 1**), and the study protocol was prospectively registered with the International Prospective Register of Systematic Reviews (PROSPERO) (https://www.crd.york.ac.uk/PROSPERO/view/CRD420261338867).

### 2.2 Eligibility criteria

Inclusion criteria: Studies were included if they met the following criteria: (i) Participants were patients diagnosed with DR. No restrictions were applied regarding nationality, race, age, or sex. (ii) A DL-based model constructed from fundus images was used for identifying DR grading. DR grading was uniformly defined according to the ICDR severity scale (8): Stage 0 – No DR: no abnormalities in the retina. Stage 1 – Mild NPDR: presence of microaneurysms (small bulges in retinal blood vessels). Stage 2 – Moderate NPDR: microaneurysms and intraretinal hemorrhages, hard and soft exudates. Stage 3 – Severe NPDR: extensive retinal blood vessel blockage, with multiple hemorrhages, venous beading, and intraretinal microvascular abnormalities. Stage 4 – PDR: new, fragile blood vessels growing into the retina and vitreous that may bleed, causing severe vision loss. (iii) Study design was a case-control, cross-sectional, or cohort study, and studies were published in English.

Exclusion criteria: Studies were excluded if they: (i) were studies in which a constructed DL-based model was not based on fundus images; (ii) were studies in which the model applied was not a DL-based model; (iii) were studies for which a full text was not available, or data were incomplete; or (iv) were conference abstracts published publicly without peer review.

### 2.3 Data source and search strategy

PubMed, Embase, Web of Science, and Cochrane Library were systematically searched up to October 28, 2025, without restrictions on region or publication year. Search strategies were formulated based on two main themes, namely ‘Diabetic Retinopathy’ and ‘Deep Learning’, combining MeSH terms and free-text terms. Boolean operators were employed to combine relevant search terms. For instance, the structure ‘(Diabetic Retinopathy[MeSH] OR free-text terms) AND (Deep Learning[MeSH] OR free-text terms)’ was adopted in PubMed. Search strategies were adjusted according to the vocabulary and syntax of each database, as provided in the **Supplementary Materials**. In addition, supplementary searches were carried out by reviewing reference lists and querying preprint platforms to minimize potential omissions (**Supplementary Material 1**).

### 2.4 Literature screening and data extraction

All retrieved records were imported into EndNote. After duplicates were removed, the titles and abstracts of the remaining records were screened to identify potentially eligible studies. Subsequently, the full texts of these studies were comprehensively reviewed to determine the final studies included. Prior to data extraction, a standardized electronic template was prepared. The extracted items included title, first author, publication year, country, study design, patient source, image source, criteria for severity measurement, number of cases per severity level, total number of cases, total number of cases in a training set, method for generating a validation set and a test set, external validation, number of cases in a validation set, number of cases in a test set, type of model, and presence or absence of a comparison with clinicians. The aforementioned literature screening and data extraction were performed by two researchers (Xin Yan and Lifen Hu), and the results were cross-checked. Any disagreements were reconciled by consulting with a third investigator (Mu Qin).

### 2.5 Risk of bias assessment

A quality assessment tool internationally recognized, Quality Assessment of Diagnostic Accuracy Studies-2 (QUADAS-2) (18), was employed to systematically assess the risk of bias and applicability of all included studies. QUADAS-2 covers four domains for risk of bias: patient selection, index test, reference standard, and flow and timing. Applicability was assessed in three domains, namely patient selection, index test, and reference standard. Each domain was rated as having ‘low risk’, ‘high risk’, or ‘unclear risk’. Risk of bias was assessed independently by two researchers (Xin Yan and Shiqi Lei), and all discrepancies were reconciled through consultation with a third investigator (Mu Qin).

### 2.6 Statistical analysis

All statistical analyses were performed with R (version 4.5.2). Since this study involved a multi−classification task based on DL, sensitivity and the misjudgment rate for each category were used to evaluate the performance of DL in discriminating among different levels of DR severity. Prior to meta−analysis, a double−arcsine transformation was applied. Model selection was based on the heterogeneity index (I²). If I² > 50%, a random−effects model was adopted. When I² < 50%, a fixed−effects model was used. P < 0.05 denoted a statistically significant difference.

## 3 Results

### 3.1 Literature screening

**Figure 1:**
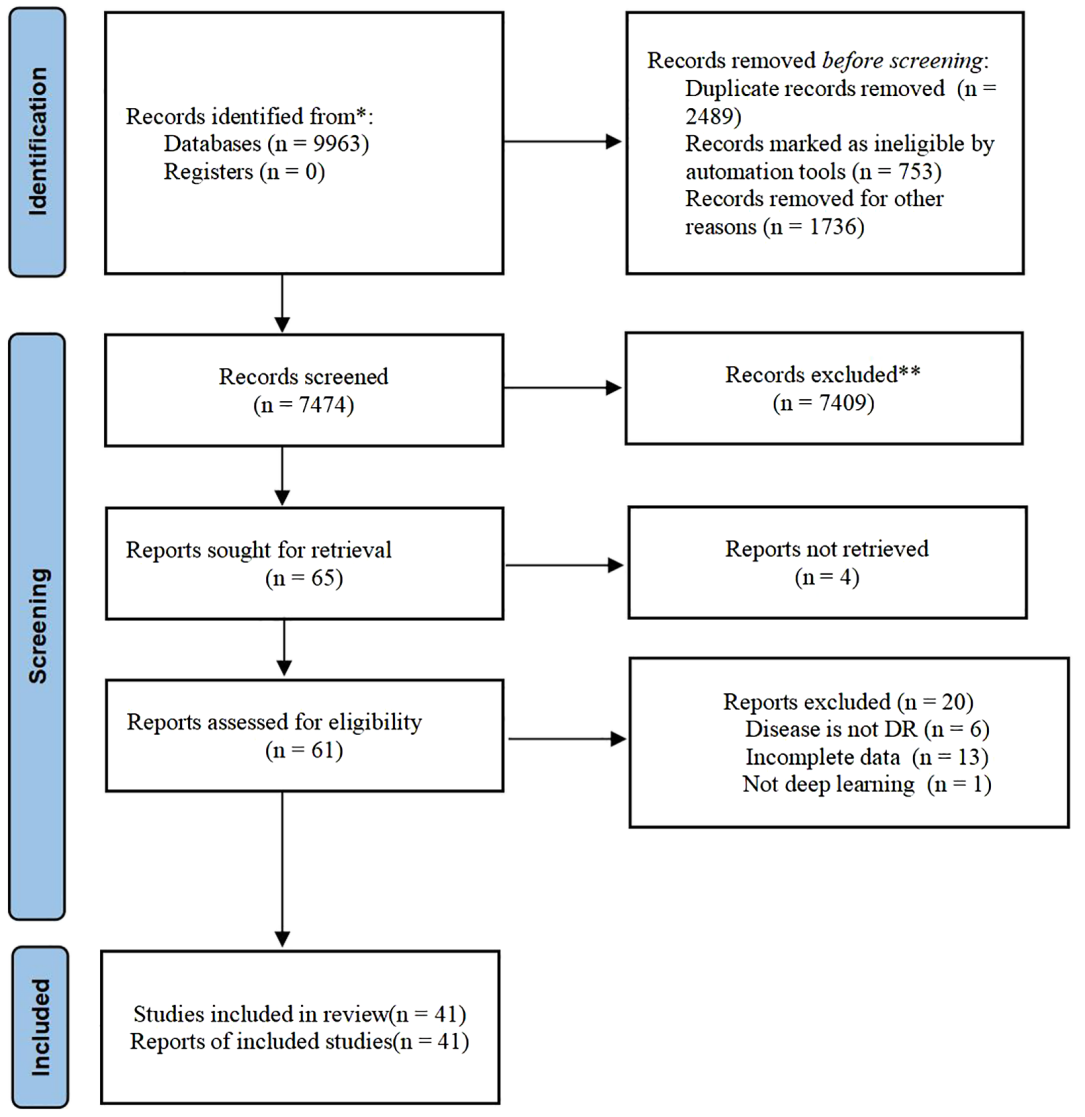
PRISMA flow diagram for literature screening.

PubMed, Embase, Web of Science, and Cochrane Library were systematically searched, and 9,963 relevant records were identified. After 2,489 duplicate records were removed via EndNote, the titles and abstracts of the remaining 7,474 articles were assessed. At this stage, 7,409 articles clearly irrelevant were excluded, and the full texts of the remaining 65 articles were reviewed. Consequently, 20 studies were excluded for not meeting the inclusion criteria. Specifically, in six studies, study subjects were not patients with DR, thirteen studies reported incomplete data, and in one study, a DL method was not employed. Ultimately, 41 studies (19–59) were included (**Figure 1**).

### 3.2 Characteristics of included studies

Totally 41 studies evaluating the diagnostic performance of DL-based models for DR grading were included. Fundus images were used as input data in all included studies, covering a variety of study designs and dataset sources. The total sample size exceeded 500,000 fundus images, with the largest single study containing approximately 1.6 million images. All studies were case−series in design. The studies originated from 16 countries, mainly India (11 studies), China (9 studies), the United States (3 studies), and Pakistan (3 studies), as well as Spain, the United Kingdom, Saudi Arabia, Malaysia, France, Colombia, Thailand, Qatar, Kuwait, Bangladesh, Romania, and Japan. The highest proportions of studies were contributed by India and China, reflecting the activity of these two countries in AI research on DR. Publicly available fundus image datasets were used in 37 studies, including EyePACS (9 studies), APTOS 2019 (12 studies), Messidor (8 studies), IDRiD (6 studies), and DDR (5 studies). Proprietary datasets from internal hospital sources were employed in 4 studies. A complete five−class grading system (stages 0–4) was adopted in 37 studies, whereas a simplified four−class system was utilized in 9 studies. Regarding image preprocessing, standardized processing was applied to input images in most studies, with common resolutions including 224×224 and 512×512, and multi−scale or cropping strategies were employed in some studies to enhance the robustness of a model. In terms of model type, a wide range of DL architectures were covered, including convolutional neural networks (CNNs) (e.g, ResNet, VGG, DenseNet, and EfficientNet), attention mechanisms and Transformer models (e.g., CoT−XNet, MVLA−Net, and Vision Transformer), and hybrid or ensemble models (e.g., HFF−Net, ADAS, and DiaRetULS−Net) frequently enhanced by feature fusion, multi−task learning, or ensemble strategies to optimize performance. Random sampling was used to split a training set and a test set in 29 studies (70.7%), with a common split ratio being 7:3 or 8:2 (training:testing). K−fold cross−validation was employed in 7 studies (17.1%), among which 5−fold cross−validation was dominant (5 studies) and 10−fold cross−validation was used in the other 2 studies. External or multi−center validation was performed in 5 studies (12.2%) to more rigorously evaluate the generalizability and clinical applicability of relevant models. Among these, independent external datasets were used for validation in 2 studies, and multi−center data were used for model testing in 3 studies. Among the studies that employed random sampling, an 8:2 split ratio was the most frequently observed (18 studies), followed by a 7:3 split ratio (11 studies). In a few studies (e.g., Sayres et al., (58)), more refined splitting methods (e.g., 7:1.5:1.5) were adopted to accommodate multi−stage validation (**Supplementary Table 1**).

### 3.3 Risk of bias assessment

**Figure 2:**
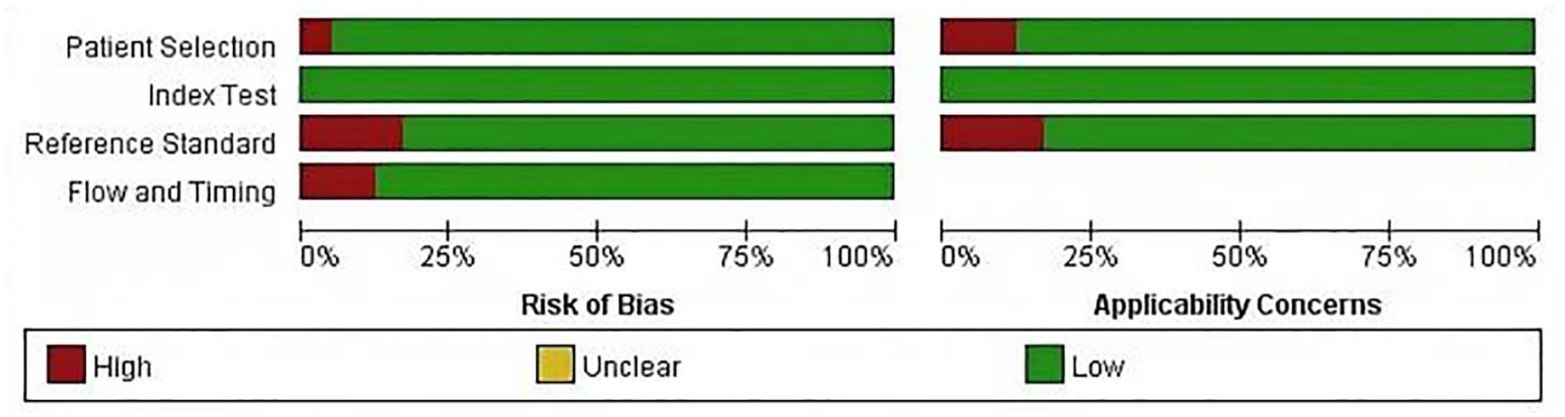
Summary plot for risk of bias assessment using QUADAS-2 for included studies.

During the overall assessment of risk of bias across all included studies, the following findings were observed. In the domain of patient selection, all included studies clearly reported the inclusion and exclusion criteria for study subjects, and all patients were enrolled based on the gold standard for DR diagnosis (the ICDR grading system). Accordingly, all studies in this domain were judged as having a ‘low risk of bias’. Fundus images were used as input data in all studies, which was consistent with the objective of our study; thus, applicability was rated as ‘low-risk’. In the domain of the conduct or interpretation of the index test, the index test was defined as an automated DR grading model based on DL. A clear classification threshold had been established in all studies before model training, and no tuning was performed using the reference standard results during model development. Consequently, all studies were rated as having a ‘low risk of bias’ in this domain. Fundus images conforming to routine clinical practice were employed in all studies, and the grading standard was consistent with the ICDR system; applicability was therefore considered ‘low-risk’. In the domain of the conduct and interpretation of the reference standard, the reference standard was expert human interpretation based on the ICDR grading system. Among the 41 studies, 33 (80.5%) explicitly reported that grading was performed by experienced ophthalmologists who were blinded to the results of the DL-based models, and these studies were assessed as having a ‘low risk of bias’. In the domain of reference standard, 8 studies (19.5%) were judged as having a ‘high risk of bias’. The main reasons for this judgment were: (i) failure to clearly describe the implementation details of the reference standard, such as whether ICDR grading of fundus images was performed independently by two or more experienced ophthalmologists; (ii) lack of specification regarding whether the graders were blinded to the diagnosis results of the DL-based models, namely, whether the model predictions were used as a reference for interpreting the reference standard; and (iii) in some studies, only the use of pre−assigned labels from publicly available datasets as the gold standard was mentioned, without verification of the reliability and consistency of these labels. The above deficiencies might have introduced measurement bias, thereby affecting the true estimation of diagnostic accuracy. In the domain of flow and timing, 4 studies (9.8%) were rated as having a ‘high risk of bias’. The main reasons comprised: (i) failure to explicitly report whether all enrolled cases were included in the final analysis, with missing cases not accounted for; (ii) unclear description of a process of data splitting, such as omission of the timing of constructing a training set, a validation set, and a test set, and whether stratified random sampling was used, which prevented from determining the presence of sample selection bias; and (iii) lack of a clear specification of the time interval between the index test (DL-based model diagnosis) and the reference standard (expert grading), especially in studies using historical data, where the potential interference of disease progression on diagnostic consistency could not be ruled out. The remaining studies clearly described the relevant procedures in the above domains, and their applicability was judged as ‘low-risk’, suggesting that the included studies were well suited for clinical application (**Figure 2**, **Supplementary Figure 1**, **Table 1**).

**Table 1:** Meta−analysis of the five−class classification task for DR detection using DL based on fundus images.

	Stage	Prediction label				
		Stage 0 (95%CI)	Stage 1 (95%CI)	Stage 2 (95%CI)	Stage 3 (95%CI)	Stage 4 (95%CI)
True label	Stage 0	95.19 (93.00-97.00)	1.67 (0.90-2.63)	1.93 (1.08-2.99)	0.01 (0.00-0.11)	0.00 (0.00-0.03)
	Stage 1	11.53 (6.71-17.39)	72.06 (62.06-81.09)	10.21 (6.86-14.11)	0.20 (0.02-0.51)	0.19 (0.02-0.46)
	Stage 2	4.68 (2.55-7.36)	3.09 (2.01-4.36)	84.33 (78.90-89.10)	2.96 (1.68-4.53)	1.20 (0.64-1.90)
	Stage 3	0.85 (0.23-1.72)	0.60 (0.13-1.27)	12.15 (8.03-16.92)	75.84 (68.42-82.57)	5.41 (3.46-7.72)
	Stage 4	1.03 (0.36-1.94)	0.86 (0.24-1.75)	6.85 (4.36-9.78)	6.69 (4.00-9.94)	78.82 (71.76-85.13)

### 3.4 Results of meta−analysis

#### 3.4.1 Five−class classification task

When stage 0 DR was diagnosed by DL, a sensitivity of 95.19% (95% CI: 93.00% – 97.00%) was observed (**Supplementary Figure 2A**). The misjudgment rate into stage 1 was 1.67% (95% CI: 0.90% – 2.63%) (**Supplementary Figure 2B**), that into stage 2 was 1.93% (95% CI: 1.08% – 2.99%) (**Supplementary Figure 2C**), that into stage 3 was 0.01% (95% CI: 0.00% – 0.11%) (**Supplementary Figure 2D**), and that into stage 4 was 0.00% (95% CI: 0.00% – 0.03%) (**Supplementary Figure 2E**).

For DL-based diagnosis of stage 1 DR, a sensitivity of 72.06% (95% CI: 62.06%–81.09%) was derived (**Supplementary Figure 3A**). Misjudgment into stage 0 occurred at a rate of 11.53% (95% CI: 6.71%–17.39%) (**Supplementary Figure 3B**), and that into stage 2 was found to be 10.21% (95% CI: 6.86%–14.11%) (**Supplementary Figure 3C**). The misjudgment rates into stages 3 and 4 were 0.20% (95% CI: 0.02%–0.51%) (**Supplementary Figure 3D**) and 0.19% (95% CI: 0.02%–0.46%) (**Supplementary Figure 3E**), respectively.

When stage 2 DR was diagnosed by DL, the sensitivity was 84.33% (95% CI: 78.90%–89.10%) (**Supplementary Figure 4A**). The misjudgment rate into stage 0 was calculated as 4.68% (95% CI: 2.55%–7.36%) (**Supplementary Figure 4B**), whereas that into stage 1 was computed at 3.09% (95% CI: 2.01%–4.36%) (**Supplementary Figure 4C**). The misjudgment rate into stage 3 was 2.96% (95% CI: 1.68%–4.53%) (**Supplementary Figure 4D**), and that into stage 4 was 1.20% (95% CI: 0.64%–1.90%) (**Supplementary Figure 4E**).

Regarding stage 3 DR diagnosed by DL, a sensitivity of 75.84% (95% CI: 68.42%–82.57%) was noted (**Supplementary Figure 5A**). The misjudgment rate into stage 0 was 0.85% (95% CI: 0.23%–1.72%) (**Supplementary Figure 5B**), and that into stage 1 was 0.60% (95% CI: 0.13%–1.27%) (**Supplementary Figure 5C**). A higher rate of 12.15% (95% CI: 8.03%–16.92%) was recorded for misjudgment into stage 2 (**Supplementary Figure 5D**), and a rate of 5.41% (95% CI: 3.46%–7.72%) was noted for that into stage 4 (**Supplementary Figure 5E**).

When stage 4 DR was diagnosed by DL, the sensitivity was 78.82% (95% CI: 71.76%–85.13%) (**Supplementary Figure 6A**). Misjudgment into stage 0 was computed at a rate of 1.03% (95% CI: 0.36%–1.94%) (**Supplementary Figure 6B**), and that into stage 1 was calculated at a rate of 0.86% (95% CI: 0.24%–1.75%) (**Supplementary Figure 6C**). The misjudgment rate into stage 2 was found to be 6.85% (95% CI: 4.36%–9.78%) (**Supplementary Figure 6D**), whereas that into stage 3 was 6.69% (95% CI: 4.00%–9.94%) (**Supplementary Figure 6E**, **Table 2**).

**Table 2:** Meta−analysis of the four−class classification task for DR detection using DL based on fundus images.

	Stage	Prediction label			
		Stage 0 (95%CI)	Stage 1 (95%CI)	Stage 2 (95%CI)	Stage 3 (95%CI)
True label	Stage 0	96.85 (90.18-99.93)	1.33 (0.02-4.13)	0.44 (0.00-1.68)	0.02 (0.00-0.22)
	Stage 1	2.85 (0.00-9.64)	92.94 (79.50-99.72)	2.09 (0.00-7.00)	0.08 (0.00-0.60)
	Stage 2	0.87 (0.00-3.67)	1.57 (0.00-5.42)	92.75 (79.31-99.61)	2.40 (0.01-7.71)
	Stage 3	0.49 (0.00-1.92)	0.63 (0.00-2.76)	3.23 (0.25-8.80)	88.19 (68.99-98.93)

#### 3.4.2 Four−class classification task

When stage 0 DR was diagnosed by DL, a sensitivity of 96.85% (95% CI: 90.18%–99.93%) was observed (**Supplementary Figure 7A**). The misjudgment rate into stage 1 was computed as 1.33% (95% CI: 0.02%–4.13%) (**Supplementary Figure 7B**), while that into stage 2 was calculated to be 0.44% (95% CI: 0.00%–1.68%) (**Supplementary Figure 7C**). Misjudgment into stage 3 was at a rate of 0.02% (95% CI: 0.00%–0.22%) (**Supplementary Figure 7D**).

For DL-based diagnosis of stage 1 DR, the sensitivity was 92.94% (95% CI: 79.50%–99.72%) (**Supplementary Figure 8A**). Misjudgment into stage 0 was found to occur at a rate of 2.85% (95% CI: 0.00%–9.64%) (**Supplementary Figure 8B**), and that into stage 2 at 2.09% (95% CI: 0.00%–7.00%) (**Supplementary Figure 8C**). A rate of 0.08% (95% CI: 0.00%–0.60%) was recorded for misjudgment into stage 3 (**Supplementary Figure 8D**).

Regarding stage 2 DR diagnosed by DL, a sensitivity of 92.75% (95% CI: 79.31%–99.61%) was achieved (**Supplementary Figure 9A**). The misjudgment rate into stage 0 was computed as 0.87% (95% CI: 0.00%–3.67%) (**Supplementary Figure 9B**), whereas that into stage 1 was 1.57% (95% CI: 0.00%–5.42%) (**Supplementary Figure 9C**). Misjudgment into stage 3 occurred at a rate of 2.40% (95% CI: 0.01%–7.71%) (**Supplementary Figure 9D**).

When stage 3 DR was diagnosed by DL, a sensitivity of 88.19% (95% CI: 68.99%–98.93%) was reported (**Supplementary Figure 10A**). Misjudgment into stage 0 was calculated as 0.49% (95% CI: 0.00%–1.92%) (**Supplementary Figure 10B**), and that into stage 1 as 0.63% (95% CI: 0.00%–2.76%) (**Supplementary Figure 10C**). A rate of 3.23% (95% CI: 0.25%–8.80%) was documented for misjudgment into stage 2 (**Supplementary Figure 10D**).

## 4 Discussion

### 4.1 Summary of evidence

In total, 41 studies were included, through which the diagnostic performance of DL for DR grading was systematically evaluated. The main findings are summarized as follows: (i) High sensitivity was demonstrated by DL in screening for ‘no DR’ (95.19% for stage 0 in the five−class classification task and 96.85% for stage 0 in the four−class classification task) and for ‘vision−threatening DR’ (78.82% for stage 4 in the five−class classification task and 88.19% for stage 3 in the four−class classification task), confirming its potential for large−scale screening and referral triage. (ii) Model performance was influenced by the granularity of grading. Specifically, in the five−class classification task, mild NPDR (stage 1) was identified with the greatest difficulty (sensitivity of 72.06%), and misjudgment occurred mainly into the adjacent stage 0 or stage 2. After the simplified four−class system was adopted, sensitivity at all grades generally increased (e.g., sensitivity at stage 1 rose to 92.94%), indicating that while overall performance was enhanced through classification simplification, the precision of grading was compromised.

### 4.2 Current status of DR grading methods

Currently, DR grading is primarily based on the ICDR or ETDRS criteria. The ICDR criteria are valued for their clinical practicality, and their nature of continuous grading renders them suitable for regression analysis in DL, thereby enhancing the accuracy of prediction (60). Despite the fact that the ETDRS criteria offer higher precision, their operation is more complex (61). Differences exist in lesion definition and severity classification between the ICDR criteria and the ETDRS criteria, and such heterogeneity may lead to inconsistencies in clinical decision−making (62). In this study, the ICDR criteria were adopted by all included studies, and comparison with the ETDRS criteria was not performed. In the future, comparative studies between these two standards should be deepened. Variations in grading criteria may cause fluctuations in algorithm performance. For instance, the model developed by Gulshan et al. achieved an AUROC of 0.991 based on the ETDRS criteria, but the threshold needs to be recalibrated when converted to the ICDR criteria (63). Wang et al. have observed a significant difference in classification accuracy between grading based on ultra−widefield fluorescein angiography and that based on ETDRS 7−field images (88.50% vs. 73.68%) (64). The selection of grading criteria directly influences clinical decision-making. Specifically, Sayres et al. have reported that AI assistance raises the sensitivity for detecting moderate or severe DR from 79.4% to 88.7%; however, the increase is uneven (58). Li et al. have found that the algorithm performed more accurately among patients with type 1 diabetes or diabetic kidney disease (sensitivity increases by 5%−8%), reflecting differences in the definition of high−risk populations across distinct criteria (65). A multimodal network (MNIIB) proposed by Song et al., which integrates different imaging features, achieves an accuracy rate of 95.9%, providing a new approach to overcoming discrepancies among different grading criteria (66). In the future, a more uniform and quantifiable grading system should be established.

### 4.3 In−depth analysis of findings

Most models included in this study were based on the five−tier ICDR grading system, whereas a few adopted a simplified four−tier system (by merging certain non−proliferative subtypes). Higher sensitivity was observed for the four−tier grading compared with the five−tier system. The reasons for this finding may be as follows: (i) Merging adjacent subtypes (e.g., mild and moderate NPDR) reduced the complexity of classification, thereby allowing the model to more readily identify clinically significant lesions. This observation is consistent with the finding of Wang et al. (2021), in which the integration of lesion−level and grade−level information increases the AUC to 0.943 (64). (ii) The number of ‘gray zone’ cases is reduced, thereby mitigating the problem of low inter−observer agreement (67). The five−tier grading system helps monitor subtle disease progression, but subtype differentiation is prone to subjective variability. The four−tier system increases the identification efficiency at key decision points (e.g., referral thresholds) and is suitable for resource−limited settings. Nevertheless, Riotto et al. (2025) have achieved an inter−observer agreement of 91% for the five−tier ICDR grading through structured consensus training, suggesting that standardized training is of vital importance (68). An analysis of misjudgment patterns revealed that most errors occurred between adjacent severity levels, consistent with the clinical challenge of distinguishing such levels, suggesting that algorithmic errors were concentrated at the ambiguous boundaries of the disease spectrum. Substantial heterogeneity was observed across studies, mainly attributable to differences in data sources, model architectures, and validation strategies, thereby affecting the generalizability of the findings. To summarize, although DL is outstanding in DR-assisted screening, fine−grained grading still needs to be optimized. In the future, it is imperative to facilitate the standardization and independent verification of relevant methodology.

### 4.4 Limitations of current DR grading criteria and future directions

Existing DR grading standards are primarily based on the morphological characteristics of blood vessels, while non−vascular factors, such as neurodegeneration and inflammation, are ignored to a great extent (4). Recent research has revealed two subtypes of PDR, namely an immune−defensive type and an endothelial cell mitochondrial dysfunction type, questioning the assumption of a single disease entity (69). With continuous advances in DL techniques, increasingly higher diagnostic performance has been achieved in the field of DR grading. However, current DL-based models mostly rely on vascular features and have limited capability to identify non−vascular factors. Moreover, they are confronted with challenges such as class imbalance and barriers to clinical translation (e.g., data privacy and liability determination) (70). In our view, future developments should be characterized by multidimensional integration and standardization improvements: (i) Multi−modal data fusion: the fusion analysis of data from emerging imaging techniques, such as ultra−widefield imaging and optical coherence tomography angiography, may become a pivotal direction. This is because these techniques provide pathological information on retinal neurodegeneration and non−vascular changes that cannot be obtained by conventional fundus photography (4). (ii) Enhanced algorithm interpretability: revealing the decision−making basis of models through attention mechanism visualization, feature inversion, and other methods may help address the issues of patient–physician trust and legal−ethical concerns associated with current ‘black−box’ algorithms (71). (iii) Expansion of application scenarios: remote screening systems supported by high−speed networks may facilitate the extension of DR grading services to regions with limited healthcare resources. (iv) Optimization of grading criteria: classification systems in the future should integrate new knowledge, such as diabetic retinal neurodegeneration, and take into account phenotype differences across future. (v) Technology innovations: the construction of cross−center standardized databases through learning privacy protection techniques, as well as the development of prediction models for therapeutic response to targeted therapies outside the VEGF pathway, may provide new insights for personalized diagnosis and treatment.

### 4.5 Real-world challenges for clinical translation and deployment barriers

The findings in this study indicate that DL-based models exhibit high diagnostic sensitivity in DR grading, particularly in identifying ‘no DR’ and ‘vision-threatening DR’. However, the translation of these models into real-world clinical settings should be viewed with caution. Currently, some studies present an overly optimistic interpretation of the clinical readiness of DL systems, while neglecting several critical deployment barriers. First, regulatory approval constitutes the primary threshold for technological implementation. Most models included in this analysis have not yet obtained approval from major regulatory authorities, nor have they undergone rigorous pre-market clinical validation. Second, the integration of DL systems into existing clinical workflows remains structurally challenging, with issues related to interoperability with electronic health record systems, real-time image acquisition and feedback latency, as well as clearly defined human−AI collaboration boundaries within screening pathways. Third, the issue of medical legal liability has yet to be properly addressed: when an erroneous grading output by a model triggers a missed or misdiagnosis, the responsible party (healthcare institution, algorithm developer, or operator) is difficult to identify, which is particularly complex in a medical system where the physician is the ultimate decision-maker. Fourth, there is generally a lack of cost−effectiveness analyses. Most studies have not reported the actual costs of model development, deployment, maintenance, or personnel training, nor have they assessed the long-term health economic benefits in real-world population screening. Accordingly, research in the future should go beyond mere technical performance validation and proactively incorporate multidisciplinary, prospective implementation studies. Clarifying regulatory pathways, workflow adaptation, liability attribution models, and health economic evaluations may lay a solid foundation for the clinical translation and widespread application of DL systems.

### 4.6 Strengths and limitations

This meta-analysis, by systematically synthesizing existing research data, provides relatively comprehensive evidence in the field of DR grading. The main strengths of this study are reflected in the cross-sectional comparative analysis conducted across multiple grading scales, thereby revealing differences between these scales and their impact on the performance of DL-based models. Furthermore, in this study, not only was the performance of existing algorithms assessed, but technical bottlenecks and future development directions were also explored in depth, thereby offering valuable references for subsequent research. Nevertheless, several limitations should be acknowledged in this study. First, substantial heterogeneity existed at the technical level among the included studies, including diversity in DL model architectures, variability in training strategies, and differing standards for image preprocessing and quality control. This heterogeneity severely affected the direct comparability between studies and the reliability of pooled results. Consequently, researchers are encouraged to perform benchmarking on standardized large−scale public datasets and to use consistent assessment metrics, thereby enhancing the comparability of results. Second, in most of the included studies, limited public datasets or single−center internal data were utilized, which may be biased in terms of demographic characteristics, type and duration of diabetes mellitus, and image acquisition equipment. As a result, the models trained on such data may perform excellently in specific populations but may suffer performance degradation when generalized to other populations, different camera models, or broader disease spectra. Although this meta-analysis described heterogeneity, it failed to completely correct such selection bias. Cross−regional and cross−ethnic external validation studies are highly encouraged to rigorously test the generalizability of models. Third, this study mainly included research based on traditional color fundus photography. Advanced studies that integrate OCTA, ultra−widefield imaging, or multimodal information, as well as frontier research exploring continuous risk scoring instead of discrete grading or identifying novel biomarkers or disease subtypes, may be insufficiently covered. This limitation influences a comprehensive insight into the most cutting−edge directions in this field. Systematic reviews in the future should continuously focus on and include AI studies based on emerging imaging technologies. In addition, the research agenda should encourage the development and validation of novel computational stratification models that go beyond the traditional ICDR framework and more effectively reflect the complex pathophysiological mechanisms of DR (e.g., incorporating neurodegeneration and inflammatory markers), along with evaluation of their clinical predictive value. Fourth, substantial variation was observed in the number of images across the included studies. Of note, few studies explicitly reported strategies for handling class imbalance (e.g., weighted loss functions, oversampling, or data augmentation). This practice may lead to severely inadequate learning for minority classes (especially severe NPDR and PDR), as reflected by significantly lower recall rates for these classes compared to majority classes. Accordingly, it is highly recommended for studies in the future to: (i) explicitly report the class distribution of the dataset and the imbalance−handling strategies employed in the Methods section; (ii) prioritize proven effective strategies such as weighted loss functions or task−specific data augmentation; and (iii) report class−specific performance metrics (particularly for minority classes) to more objectively assess the ability of a model to recognize rare but severe lesions in real−world clinical scenarios.

## 5 Conclusion

This study confirms that DL-based automated systems are highly effective for DR screening and initial stratification. Notably, the high sensitivity observed in identifying referable lesions (e.g., proliferative DR) facilitates efficient triage, particularly in resource-limited settings. Nevertheless, current technology has not yet fully matched the proficiency of clinical experts in nuanced grading, especially in differentiating between early NPDR subtypes. Model performance is profoundly influenced by the granularity of the grading criteria. While simplified classification improves overall sensitivity, it may inadvertently mask subtle indicators of disease progression. Future research should prioritize the following avenues: (i) constructing high-quality, standardized, and multicenter annotated datasets to mitigate inherent biases; (ii) developing novel algorithms that integrate multimodal imaging (e.g., OCTA) and pathophysiological biomarkers (e.g., neurodegeneration and inflammation), thereby transcending the current grading framework limited to vascular morphology; (iii) conducting prospective clinical studies to evaluate the clinical efficacy, cost-effectiveness, and impact on patient outcomes following the integration of AI systems into real-world clinical workflows; and (iv) establishing unified standards for AI reporting and performance evaluation to enhance the comparability and reproducibility of results. The profound synergy between deep learning and clinical insight is poised to drive DR management toward enhanced precision, efficiency, and accessibility.

## Data Availability

The original contributions presented in the study are included in the article/**Supplementary Material**. Further inquiries can be directed to the corresponding authors.

## Supplementary material

Supplementary Figure 1 Detailed plot for risk of bias assessment using QUADAS-2 for included studies.

Supplementary Figure 2 **(A)** Forest plot for sensitivity for diagnosing stage 0 DR based on DL. **(B)** Stage 0 DR diagnosed by DL: forest plot for misjudgment rate into stage 1. **(C)** Stage 0 DR diagnosed by DL: forest plot for misjudgment rate into stage 2. **(D)** Stage 0 DR diagnosed by DL: forest plot for misjudgment rate into stage 3. **(E)** Stage 0 DR diagnosed by DL: forest plot for misjudgment rate into stage 4.

Supplementary Figure 3 **(A)** Forest plot for sensitivity for diagnosing stage 1 DR based on DL. **(B)** Stage 1 DR diagnosed by DL: forest plot for misjudgment rate into stage 0. **(C)** Stage 1 DR diagnosed by DL: forest plot for misjudgment rate into stage 2. **(D)** Stage 1 DR diagnosed by DL: forest plot for misjudgment rate into stage 3. **(E)** Stage 1 DR diagnosed by DL: forest plot for misjudgment rate into stage 4.

Supplementary Figure 4 **(A)** Forest plot for sensitivity for diagnosing stage 2 DR based on DL. **(B)** Stage 2 DR diagnosed by DL: forest plot for misjudgment rate into stage 0. **(C)** Stage 2 DR diagnosed by DL: forest plot for misjudgment rate into stage 1. **(D)** Stage 2 DR diagnosed by DL: forest plot for misjudgment rate into stage 3. **(E)** Stage 2 DR diagnosed by DL: forest plot for misjudgment rate into stage 4.

Supplementary Figure 5 **(A)** Forest plot for sensitivity for diagnosing stage 3 DR based on DL. **(B)** Stage 3 DR diagnosed by DL: forest plot for misjudgment rate into stage 0. **(C)** Stage 3 DR diagnosed by DL: forest plot for misjudgment rate into stage 1. **(D)** Stage 3 DR diagnosed by DL: forest plot for misjudgment rate into stage 2. **(E)** Stage 3 DR diagnosed by DL: forest plot for misjudgment rate into stage 4.

Supplementary Figure 6 **(A)** Forest plot for sensitivity for diagnosing stage 4 DR based on DL. **(B)** Stage 4 DR diagnosed by DL: forest plot for misjudgment rate into stage 0. **(C)** Stage 4 DR diagnosed by DL: forest plot for misjudgment rate into stage 1. **(D)** Stage 4 DR diagnosed by DL: forest plot for misjudgment rate into stage 2. **(E)** Stage 4 DR diagnosed by DL: forest plot for misjudgment rate into stage 3.

Supplementary Figure 7 **(A)** Forest plot for sensitivity for diagnosing stage 0 DR based on DL. **(B)** Stage 0 DR diagnosed by DL: forest plot for misjudgment rate into stage 1. **(C)** Stage 0 DR diagnosed by DL: forest plot for misjudgment rate into stage 2. **(D)** Stage 0 DR diagnosed by DL: forest plot for misjudgment rate into stage 3.

Supplementary Figure 8 **(A)** Forest plot for sensitivity for diagnosing stage 1 DR based on DL. **(B)** Stage 1 DR diagnosed by DL: forest plot for misjudgment rate into stage 0. **(C)** Stage 1 DR diagnosed by DL: forest plot for misjudgment rate into stage 2. **(D)** Stage 1 DR diagnosed by DL: forest plot for misjudgment rate into stage 3.

Supplementary Figure 9 **(A)** Forest plot for sensitivity for diagnosing stage 2 DR based on DL. **(B)** Stage 2 DR diagnosed by DL: forest plot for misjudgment rate into stage 0. **(C)** Stage 2 DR diagnosed by DL: forest plot for misjudgment rate into stage 1. **(D)** Stage 2 DR diagnosed by DL: forest plot for misjudgment rate into stage 3.

Supplementary Figure 10 **(A)** Forest plot for sensitivity for diagnosing stage 3 DR based on DL. **(B)** Stage 3 DR diagnosed by DL: forest plot for misjudgment rate into stage 0. **(C)** Stage 3 DR diagnosed by DL: forest plot for misjudgment rate into stage 1. **(D)** Stage 3 DR diagnosed by DL: forest plot for misjudgment rate into stage 2.
