# Supplementary figures and images for: Diagnostic accuracy and clinical performance of deep learning models for grading diabetic retinopathy: a systematic review and meta-analysis
Source: Front Endocrinol (Lausanne). 2026 Jul 15;17:1853785. doi: 10.3389/fendo.2026.1853785 (PMC13414145; doi:10.3389/fendo.2026.1853785)

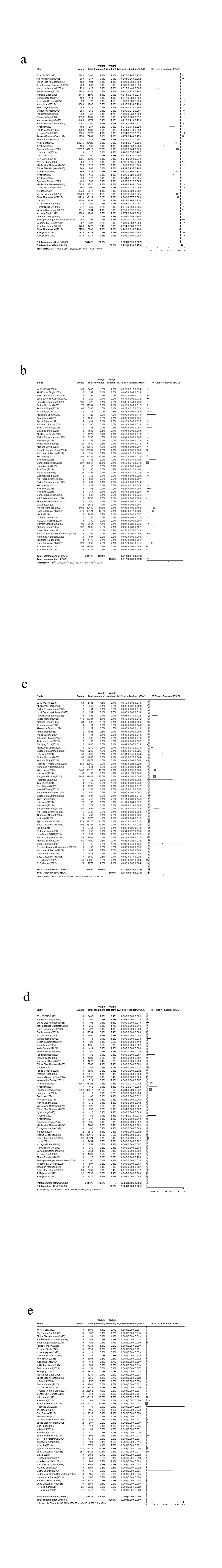

Supplement: Supplementary Figure 1 — Detailed plot for risk of bias assessment using QUADAS-2 for included studies. [file Image1.tif]

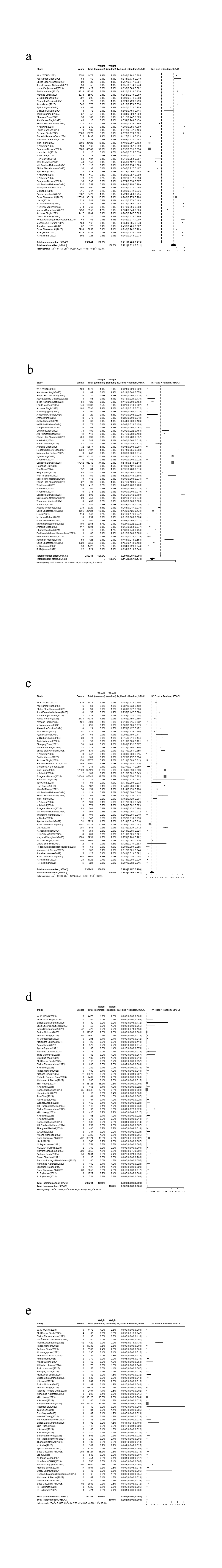

Supplement: Supplementary Figure 2 — (A) Forest plot for sensitivity for diagnosing stage 0 DR based on DL. (B) Stage 0 DR diagnosed by DL: forest plot for misjudgment rate into stage 1. (C) Stage 0 DR diagnosed by DL: forest plot for misjudgment rate into stage 2. (D) Stage 0 DR diagnosed by DL: forest plot for misjudgment rate into stage 3. (E) Stage 0 DR diagnosed by DL: forest plot for misjudgment rate into stage 4. [file Image2.tif]

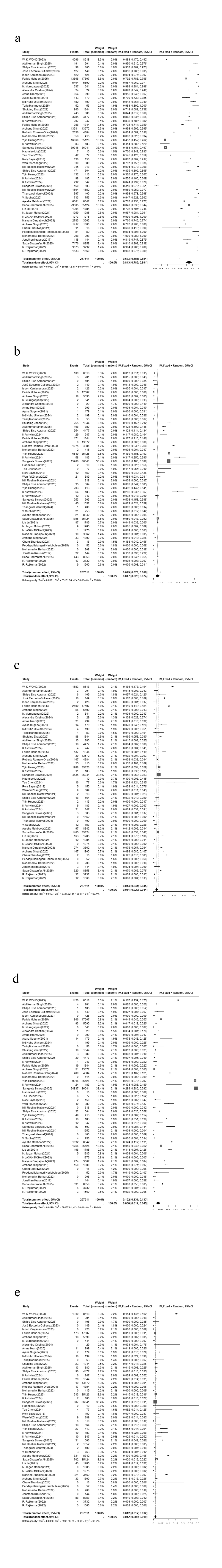

Supplement: Supplementary Figure 3 — (A) Forest plot for sensitivity for diagnosing stage 1 DR based on DL. (B) Stage 1 DR diagnosed by DL: forest plot for misjudgment rate into stage 0. (C) Stage 1 DR diagnosed by DL: forest plot for misjudgment rate into stage 2. (D) Stage 1 DR diagnosed by DL: forest plot for misjudgment rate into stage 3. (E) Stage 1 DR diagnosed by DL: forest plot for misjudgment rate into stage 4. [file Image3.tif]

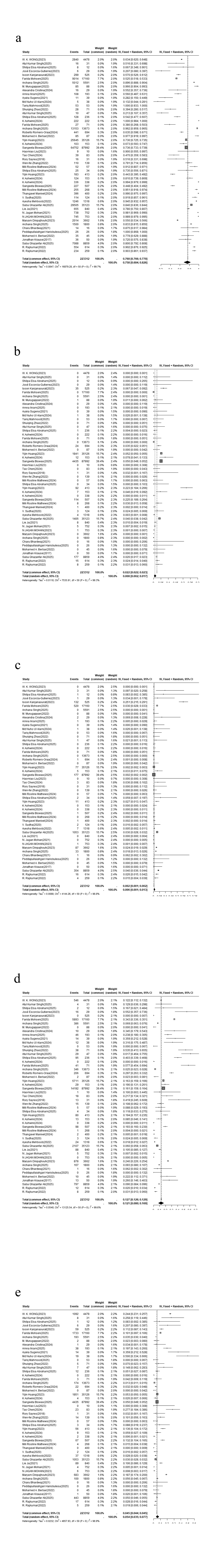

Supplement: Supplementary Figure 4 — (A) Forest plot for sensitivity for diagnosing stage 2 DR based on DL. (B) Stage 2 DR diagnosed by DL: forest plot for misjudgment rate into stage 0. (C) Stage 2 DR diagnosed by DL: forest plot for misjudgment rate into stage 1. (D) Stage 2 DR diagnosed by DL: forest plot for misjudgment rate into stage 3. (E) Stage 2 DR diagnosed by DL: forest plot for misjudgment rate into stage 4. [file Image4.tif]

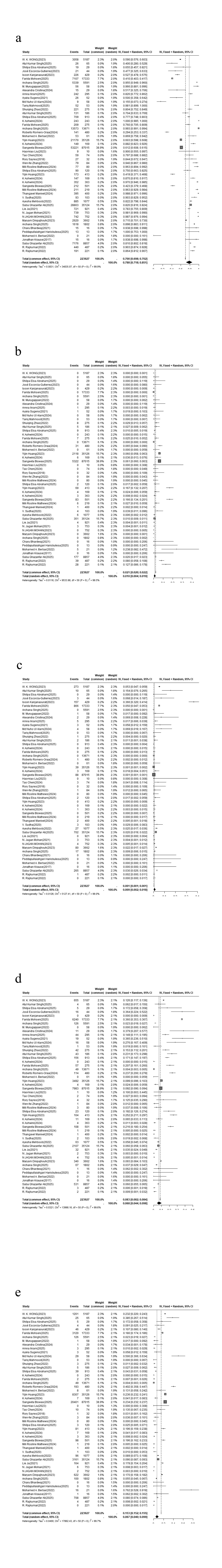

Supplement: Supplementary Figure 5 — (A) Forest plot for sensitivity for diagnosing stage 3 DR based on DL. (B) Stage 3 DR diagnosed by DL: forest plot for misjudgment rate into stage 0. (C) Stage 3 DR diagnosed by DL: forest plot for misjudgment rate into stage 1. (D) Stage 3 DR diagnosed by DL: forest plot for misjudgment rate into stage 2. (E) Stage 3 DR diagnosed by DL: forest plot for misjudgment rate into stage 4. [file Image5.tif]

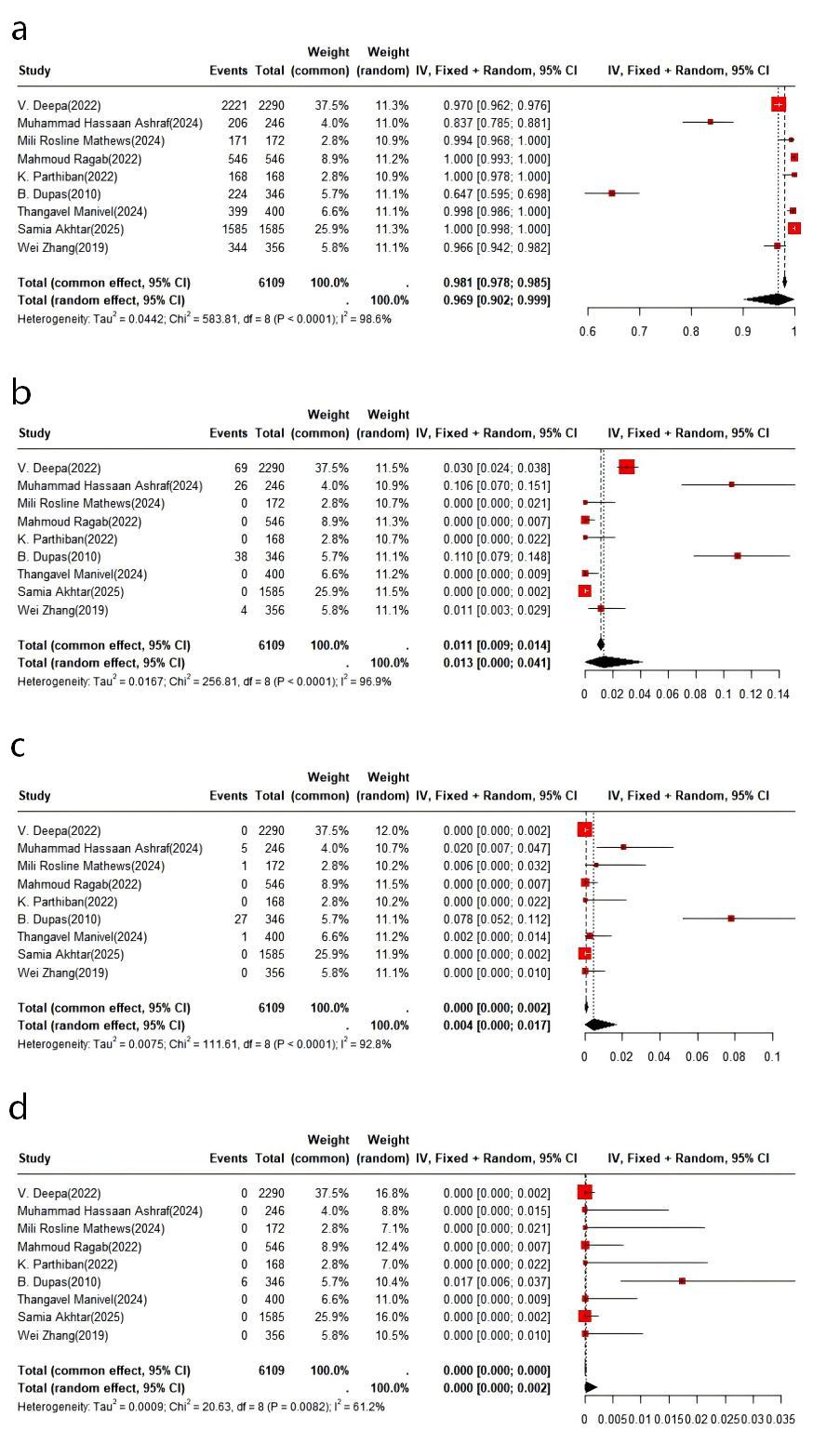

Supplement: Supplementary Figure 6 — (A) Forest plot for sensitivity for diagnosing stage 4 DR based on DL. (B) Stage 4 DR diagnosed by DL: forest plot for misjudgment rate into stage 0. (C) Stage 4 DR diagnosed by DL: forest plot for misjudgment rate into stage 1. (D) Stage 4 DR diagnosed by DL: forest plot for misjudgment rate into stage 2. (E) Stage 4 DR diagnosed by DL: forest plot for misjudgment rate into stage 3. [file Image6.jpeg]

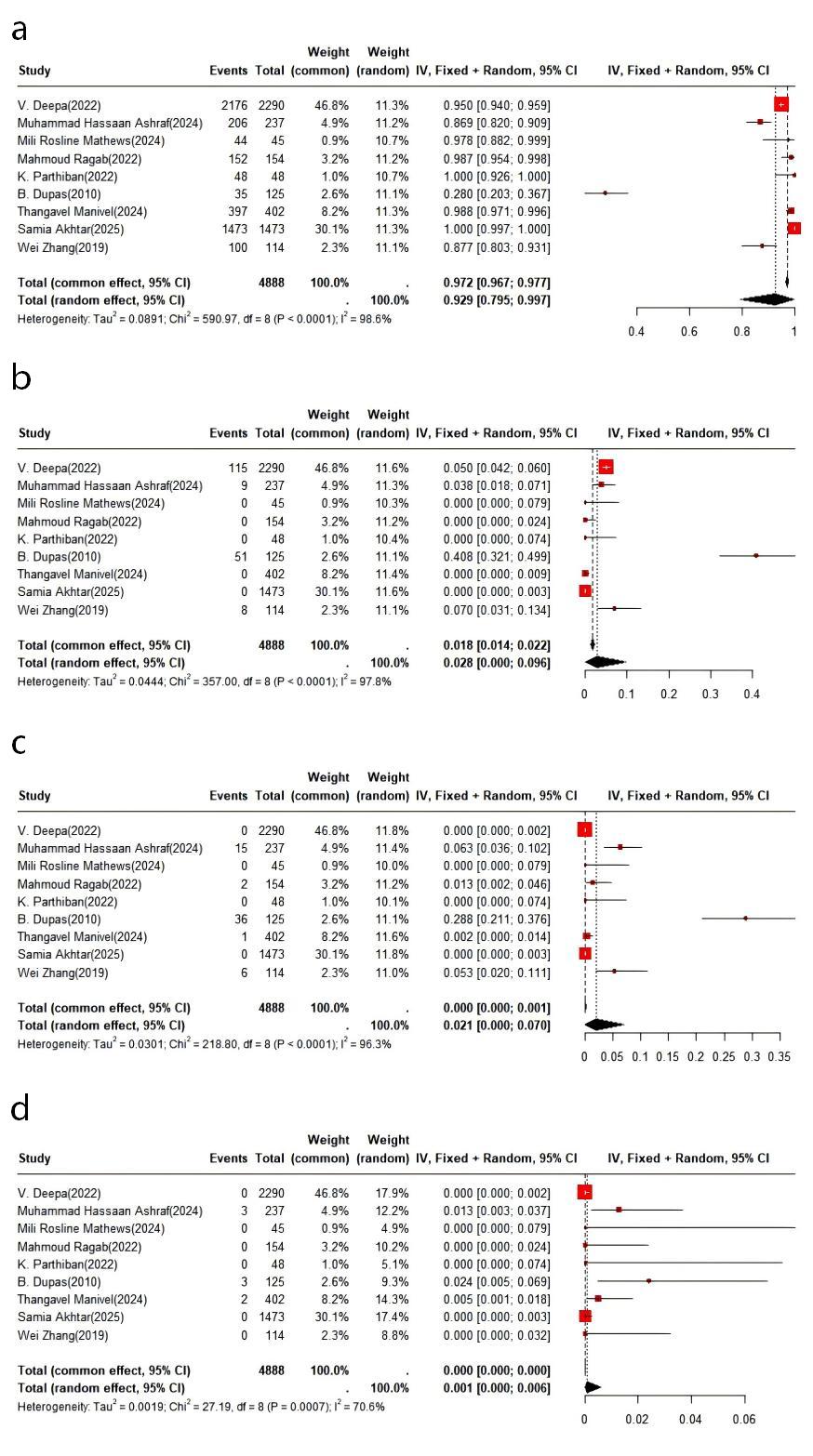

Supplement: Supplementary Figure 7 — (A) Forest plot for sensitivity for diagnosing stage 0 DR based on DL. (B) Stage 0 DR diagnosed by DL: forest plot for misjudgment rate into stage 1. (C) Stage 0 DR diagnosed by DL: forest plot for misjudgment rate into stage 2. (D) Stage 0 DR diagnosed by DL: forest plot for misjudgment rate into stage 3. [file Image7.jpeg]

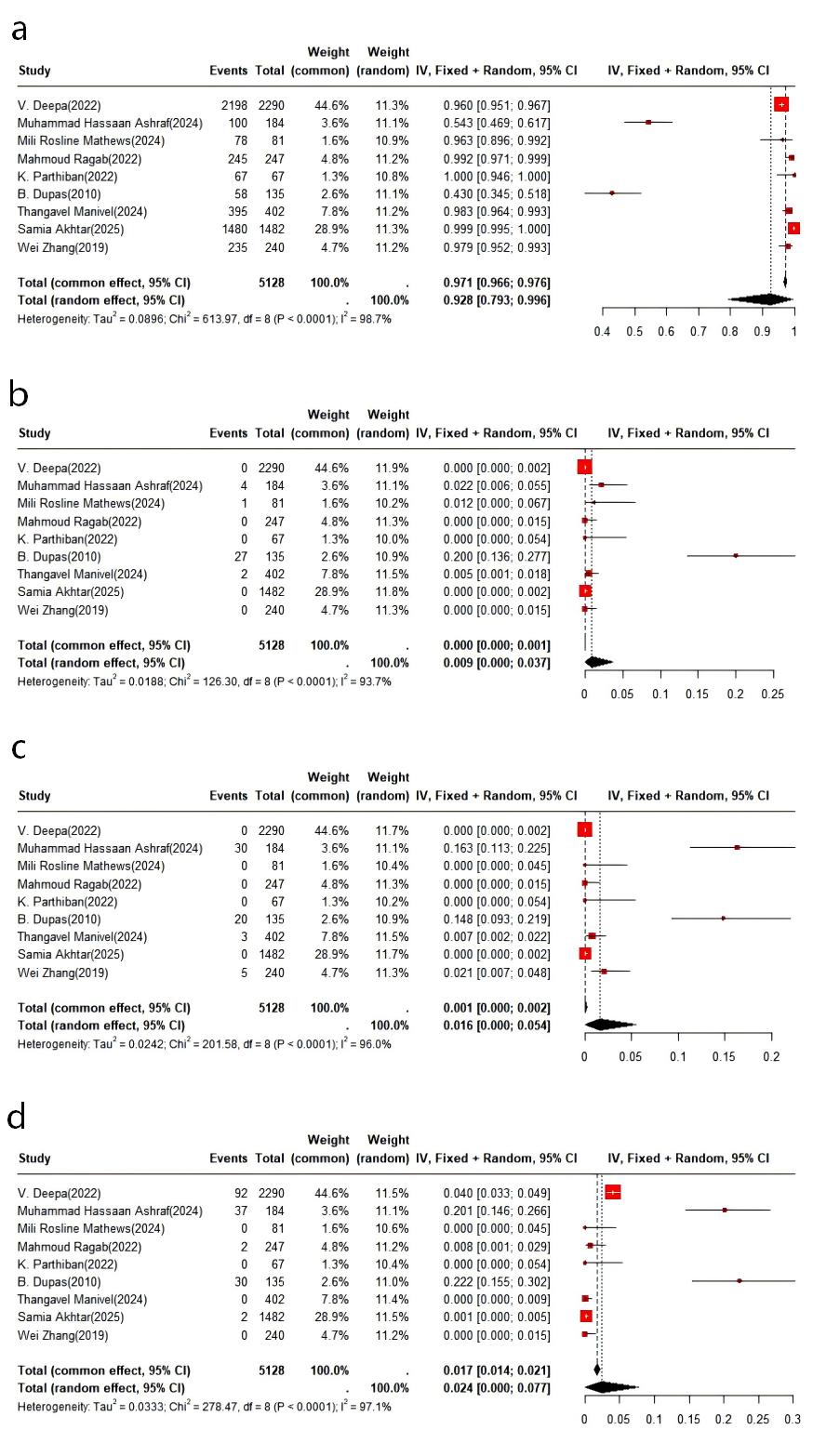

Supplement: Supplementary Figure 8 — (A) Forest plot for sensitivity for diagnosing stage 1 DR based on DL. (B) Stage 1 DR diagnosed by DL: forest plot for misjudgment rate into stage 0. (C) Stage 1 DR diagnosed by DL: forest plot for misjudgment rate into stage 2. (D) Stage 1 DR diagnosed by DL: forest plot for misjudgment rate into stage 3. [file Image8.jpeg]

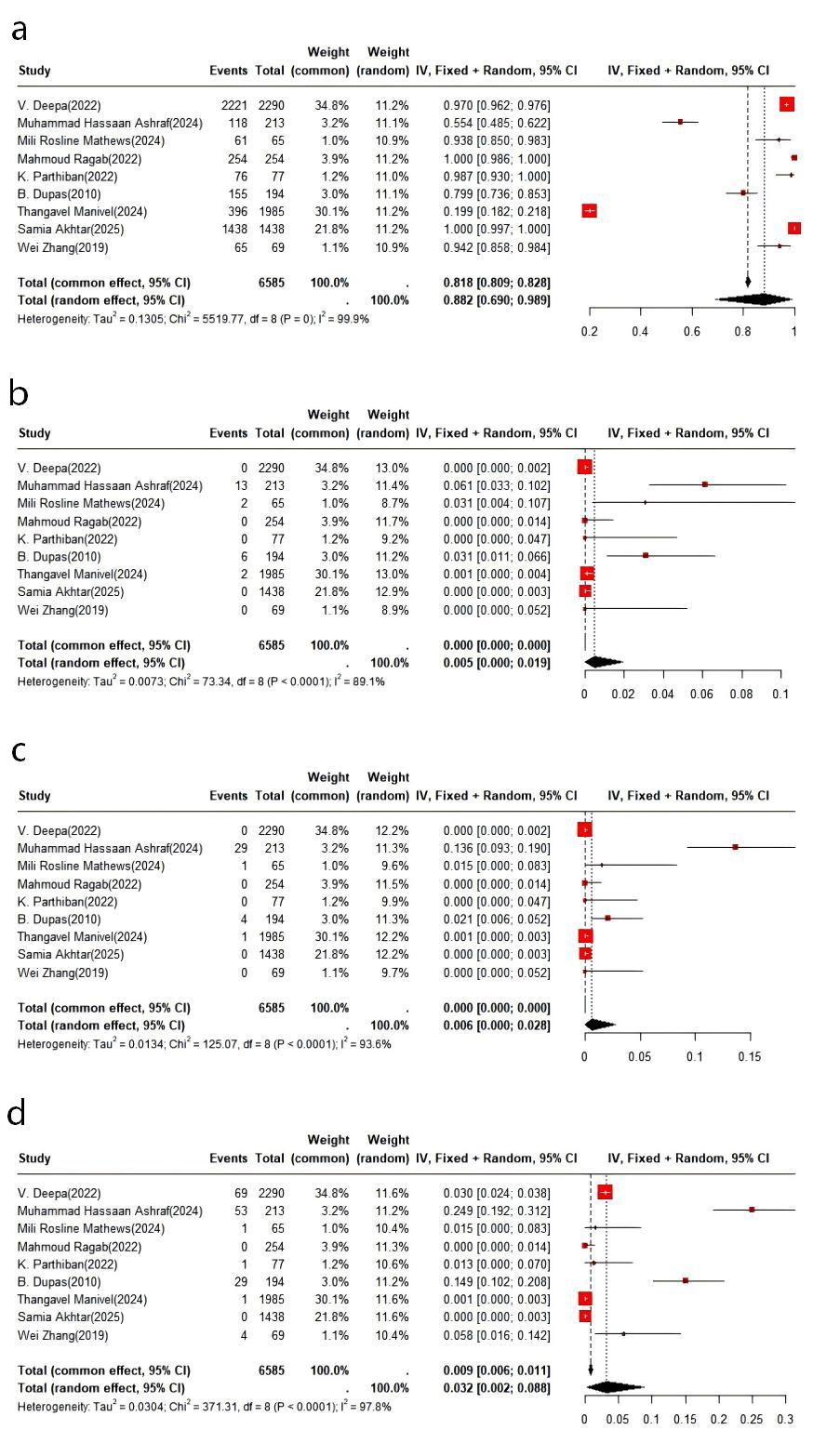

Supplement: Supplementary Figure 9 — (A) Forest plot for sensitivity for diagnosing stage 2 DR based on DL. (B) Stage 2 DR diagnosed by DL: forest plot for misjudgment rate into stage 0. (C) Stage 2 DR diagnosed by DL: forest plot for misjudgment rate into stage 1. (D) Stage 2 DR diagnosed by DL: forest plot for misjudgment rate into stage 3. [file Image9.jpeg]

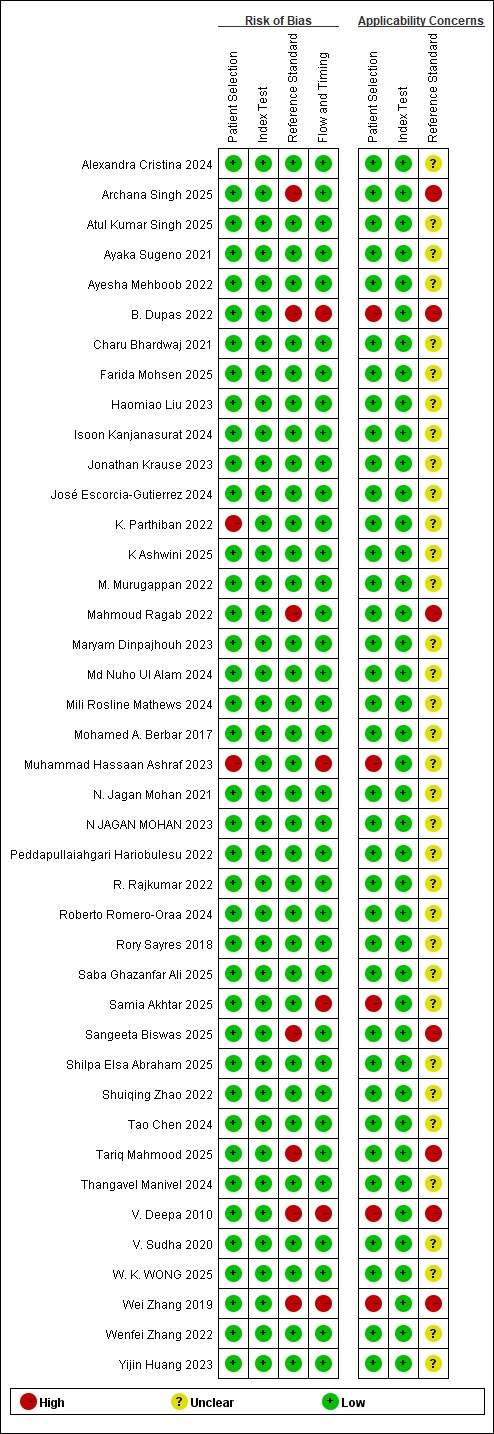

Supplement: Supplementary Figure 10 — (A) Forest plot for sensitivity for diagnosing stage 3 DR based on DL. (B) Stage 3 DR diagnosed by DL: forest plot for misjudgment rate into stage 0. (C) Stage 3 DR diagnosed by DL: forest plot for misjudgment rate into stage 1. (D) Stage 3 DR diagnosed by DL: forest plot for misjudgment rate into stage 2. [file Image10.jpeg]
