# Supplementary material for: Diagnostic accuracy and clinical performance of deep learning models for grading diabetic retinopathy: a systematic review and meta-analysis
Source: Front Endocrinol (Lausanne). 2026 Jul 15;17:1853785. doi: 10.3389/fendo.2026.1853785 (PMC13414145; doi:10.3389/fendo.2026.1853785)
Supplement: Supplementary file 11 [file DataSheet1.pdf]

1.PubMed

Step	Search term
#1	Diabetic Retinopathy［MeSH］
#2	Diabetic Retinopathy OR Diabetic Retinopathies OR diabetes mellitus retinopathy OR diabetes retinopathy OR diabetic retinitis OR retinopathia diabetica［Title/Abstract］
#3	#1 OR #2
#4	Deep Learning［MeSH］
#5	deep learning OR Transfer Learning OR Ensemble Learning OR artificial intelligence OR Hierarchical Learning OR Transfer Learning OR Machine Learning OR Neural Network* OR Deep Network* OR computer-aided diagnosis OR Generative Adversarial Network OR CNN OR AlexNet OR VGGNet OR ResNet OR GoogLeNet OR DenseNet OR Inception V3 OR MobileNet OR RNN OR LSTM OR VGG-11 OR VGG-13 OR VGG-16 OR VGG-19 OR VGG11 OR VGG13 OR VGG16 OR VGG19 OR ResNet50 OR ResNet101 OR ResNet34 OR ResNet18［Title/Abstract］
#6	#4 OR #5
#7	#3 AND #6

2.Embase

Step	Search term
#1	Diabetic Retinopathy［exp］
#2	Diabetic Retinopathy OR Diabetic Retinopathies OR diabetes mellitus retinopathy OR diabetes retinopathy OR diabetic retinitis OR retinopathia diabetica［Title/Abstract］
#3	#1 OR #2
#4	Deep Learning［exp］
#5	deep learning OR Transfer Learning OR Ensemble Learning OR artificial intelligence OR Hierarchical Learning OR Transfer Learning OR Machine Learning OR Neural Network* OR Deep Network* OR computer-aided diagnosis OR Generative Adversarial Network OR CNN OR AlexNet OR VGGNet OR ResNet OR GoogLeNet OR DenseNet OR Inception V3 OR MobileNet OR RNN OR LSTM OR VGG-11 OR VGG-13 OR VGG-16 OR VGG-19 OR VGG11 OR VGG13 OR VGG16 OR VGG19 OR ResNet50 OR ResNet101 OR ResNet34 OR ResNet18［Title/Abstract］
#6	#4 OR #5
#7	#3 AND #6

3.Cocrhane library

Step	Search term
#1	Diabetic Retinopathy［MeSH］
#2	Diabetic Retinopathy OR Diabetic Retinopathies OR diabetes mellitus retinopathy OR diabetes retinopathy OR diabetic retinitis OR retinopathia diabetica［Title/Abstract］
#3	#1 OR #2
#4	Deep Learning［MeSH］
#5	deep learning OR Transfer Learning OR Ensemble Learning OR artificial intelligence

	OR Hierarchical Learning OR Transfer Learning OR Machine Learning OR Neural Network* OR Deep Network* OR computer-aided diagnosis OR Generative Adversarial Network OR CNN OR AlexNet OR VGGNet OR ResNet OR GoogLeNet OR DenseNet OR Inception V3 OR MobileNet OR RNN OR LSTM OR VGG-11 OR VGG-13 OR VGG-16 OR VGG-19 OR VGG11 OR VGG13 OR VGG16 OR VGG19 OR ResNet50 OR ResNet101 OR ResNet34 OR ResNet18［Title/Abstract］
#6	#4 OR #5
#7	#3 AND #6

4.Web of science

Step	Search term
#1	Diabetic Retinopathy OR Diabetic Retinopathies OR diabetes mellitus retinopathy OR diabetes retinopathy OR diabetic retinitis OR retinopathia diabetica［Topic］
#2	deep learning OR Transfer Learning OR Ensemble Learning OR artificial intelligence OR Hierarchical Learning OR Transfer Learning OR Machine Learning OR Neural Network* OR Deep Network* OR computer-aided diagnosis OR Generative Adversarial Network OR CNN OR AlexNet OR VGGNet OR ResNet OR GoogLeNet OR DenseNet OR Inception V3 OR MobileNet OR RNN OR LSTM OR VGG-11 OR VGG-13 OR VGG-16 OR VGG-19 OR VGG11 OR VGG13 OR VGG16 OR VGG19 OR ResNet50 OR ResNet101 OR ResNet34 OR ResNet18［Topic］
#3	#1 AND #2
